# Pacemaker Malfunction During Passive Proton Beam Therapy for Localized Prostate Cancer: Case Reports and a Literature Review

**DOI:** 10.7759/cureus.46223

**Published:** 2023-09-29

**Authors:** Tsukasa Yoshida, Shigeyuki Murayama, Kazuaki Yasui, Tetsuya Tomida, Atsushi Urikura

**Affiliations:** 1 Department of Diagnostic Radiology, Shizuoka Cancer Center, Shizuoka, JPN; 2 Department of Radiation and Proton Therapy, Shizuoka Cancer Center, Shizuoka, JPN; 3 Department of Radiological Technology, Radiological Diagnosis, National Cancer Center Hospital, Tokyo, JPN

**Keywords:** radiotherapy (rt), pacemaker, cardiac implantable devices, proton therapy, prostate cancer

## Abstract

We report two cases of pacemaker malfunction occurring during proton beam therapy (PBT) for localized prostate cancer treatment. The first case involved mode changes in the pacemaker, while the second exhibited prolongation of the RR interval. Remarkably, both cases did not manifest significant clinical changes. Our findings indicate that careful consideration should be given to passive PBT in patients with localized prostate cancer who have pacemakers, like the considerations in patients with thoracic and abdominal cancers. Moreover, our report highlights the importance of recognizing potential cardiac implantable electronic devices malfunction in various PBT scenarios.

## Introduction

Interactions with cardiac implantable electronic devices (CIEDs), including pacemakers, are known to occur with proton beam therapy (PBT) [1]. Secondary neutrons induced by proton beam therapy can lead to the malfunction of CIEDs. Specifically, the mechanism of the malfunction is a single-event upset caused by the secondary neutron [2]. Grant et al. reported that unrecoverable reset induced by the secondary neutrons required the CIEDs replacement (2 out of 15) [3]. According to a recent publication from the American Association of Physicists in Medicine Task Group 203, PBT is considered a high-risk treatment for patients with CIEDs and requires weekly electrocardiogram monitoring [4].

Secondary neutrons associated with proton therapy affect CIEDs even when it is located outside the treatment field [5]. To date, some researchers have reported CIED malfunctions during PBT for thoracic and abdominal tumors [6-8]. Furthermore, a retrospective multi-institutional study reported that CIED malfunction during PBT occurred in the treatment site from the head and neck to the abdomen [9].

To our knowledge, no study has reported CIED malfunctions during PBT in cases where the treatment isocenter is distant from the pacemaker location, such as localized prostate cancer. Herein, we report two cases of pacemaker malfunction during PBT in patients with localized prostate cancer and briefly review previous literature.

## Case presentation

Patient characteristics

Four patients with pacemakers underwent PBT for the treatment of localized prostate cancer during the period. Table 1 shows the patient characteristics in our institution. The incidence of pacemaker malfunction was 50% (2/4) during PBT.

**Table 1:** Characteristics of patients with pacemaker treated with PBT for prostate cancer Distance was measured between the treatment isocenter and pacemaker generator OMI, old myocardial infarction; VF, ventricular fibrillation; SSS, sick sinus syndrome; AV block, atrioventricular block; Lt, left; DDDR, dual-chamber rate-adaptive pacing; DDD: dual-chamber pacing; AAIR; atrial-sensing inhibited-response rate-adaptive pacing; VVI, ventricular pacing

Age	Disease	Distance (cm)	Location	Manufacturer	Mode	Malfunction
78	OMI, VF	56	Lt. subclavicle	Medtronic	DDDR	None
67	SSS	54	Lt. subclavicle	Biotronik	DDD	Yes
64	SSS	55	Lt. subclavicle	Medtronic	AAIR⇔DDDR	Yes
75	SSS, AV block	54	Lt. subclavicle	St. Jude Medical	VVI	None

Case 1

A 67-year-old man was diagnosed with localized prostate cancer (T2cN0M0). The prescription dose was 74 Gy (relative biological effectiveness or RBE) in 37 fractions of 220 MeV passive proton beams. He had previously undergone a pacemaker implantation for sick sinus syndrome (Edora8, Biotronik, Oregon, USA) below the left clavicle. The treatment isocenter was approximately 54 cm distant from the position of the pacemaker generator. Electrocardiogram (ECG) monitoring was performed during PBT every fraction in the presence of a medical engineer. The pacemaker was found to be switched to “back-up mode” in the 8th fraction. Thereafter, the mode change of the pacemaker was found to occur from DDD (60 bpm) to DDI (70 bpm) according to the log analysis. In addition, no remarkable changes were reported in the medical examination according to a cardiologist.

Case 2

A 64-year-old man diagnosed with localized prostate cancer (T2aN0M0) was prescribed 74 Gy (RBE) in 37 fractions of 220 MeV passive proton beams. He had previously undergone a pacemaker implantation for the management of sick sinus syndrome (Adapta ADDR01, Medtronic, Minnesota, USA) below the left clavicle. The treatment isocenter was approximately 55 cm distant from the pacemaker generator. ECG monitoring was performed during PBT every fraction in the presence of a medical engineer. In the 3rd, 5th, 8th, and 28th fractions, prolongation of RR intervals occurred during PBT. The medical engineer confirmed that oversensing due to the noise from the proton beam caused the prolongation of RR intervals. Moreover, no remarkable changes were reported in the medical examination by a cardiologist.

## Discussion

To the best of our knowledge, this is the first report of pacemaker malfunctions in patients who underwent PBT for the treatment of localized prostate cancer. Previous studies have reported CIED malfunction during PBT (Table 2). Oshiro et al. reported that PBT caused pacemaker malfunction, such as changes in pacing rate and pulse patterns [6]. Gomez et al. reported that the incidence of CIED resets was observed in patients who had PBT placed in the thorax and liver [7]. Ueyama et al. reported that the pacemaker reset occurred during PBT placed in patients with lung and pancreatic cancer [8].

**Table 2:** Published works of interactions between CIED and PBT CIED, cardiac implantable electronic devices; PBT, proton beam therapy; ICP, implantable cardiac pacemaker; ICD, implantable cardioverter defibrillator; Gy (RBE), gray (relative biological effectiveness)

Author	Year	CIED	Treatment site	Prescribed dose	Outcome
Oshiro [6]	2008	ICP	Lung, liver	33–77 Gy (RBE) Passive scattering	Changes in pulse rate and pulse patterns
Gomez [7]	2013	ICP, ICD	Thorax Liver	50.4–87.5 Gy (RBE) Passive scattering	Device reset
Ueyama [8]	2016	ICP	Lung, Pancreas	50–60 Gy (RBE) Passive scattering	Device reset

**Figure 1:**
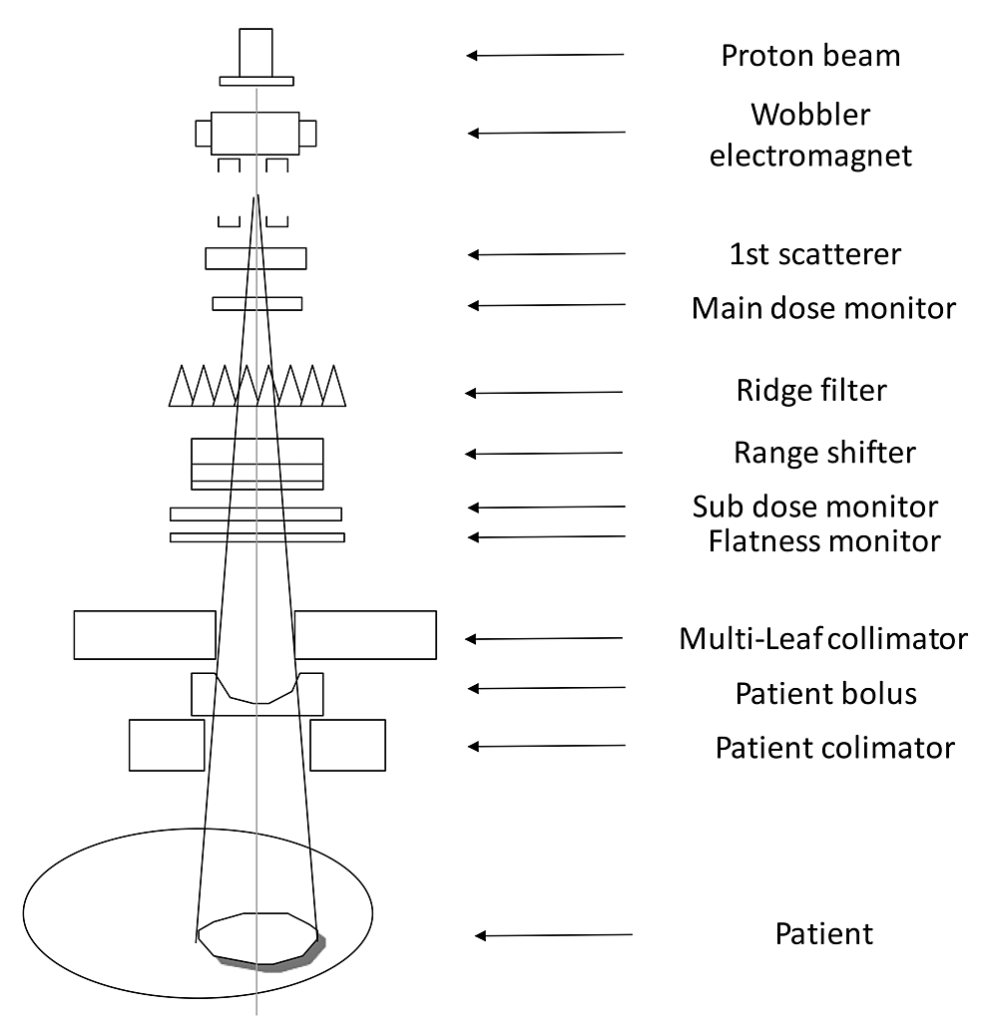
Schematic of the passive scattering proton beam delivery system The proton beam is delivered to patients through the beam modulators and collimators Secondary neutrons are generated from several scattering materials (1st scatterer, ridge filter, and range shifter) and collimators (multi-leaf and patient collimators) Image Credits: Tsukasa Yoshida

The possible explanation for the pacemaker malfunction in our case is secondary neutrons released outside the treatment field, as described in previous literature [10]. The main sources of secondary neutrons are several scattering materials (1st scatterer, ridge filter, and range shifter) and collimators (multi-leaf collimator and patient collimator) in our proton therapy system (Figure 1). Secondary neutrons are more frequently generated using passive scattering systems than pencil beam scanning systems. Moreover, secondary neutrons from the passive scattering system are more commonly detected than those from the pencil beam scanning system at a position distant from the beam isocenter [4].

According to previous studies, the passive scattering system has been used in many institutions (Table 2). One institution reported that pacemaker malfunction is not observed in prostate treatment where the treatment field is >50 cm distant from the pacemaker generator [4]. However, the incidence of pacemaker malfunction in our institution was 50% although the treatment isocenter is approximately >50 cm apart from the pacemaker generator in both cases. Our result indicates that PBT using the passive scattering system can potentially cause interaction with pacemakers even if pacemaker locations are distant from the treatment isocenter.

The incidence rate of CIED malfunction in X-ray therapy is reported by Grant et al. [3]. They reported the incidence rate of CIED malfunction was 35% in pelvic treatments using X-rays. Compared to their reports, the incidence rate in our case was relatively high. However, it is preferred to consider the incidence of pacemaker malfunctions due to the relatively small sample size of patients with pacemakers who underwent the passive PBT in our report. Therefore, further investigation is warranted to discuss the incidence of the CIEDs malfunction.

## Conclusions

We reported two cases of pacemaker malfunction during PBT for the treatment of localized prostate cancer. Our report indicates that pacemaker malfunction during PBT may occur at the treatment isocenter much distant from the pacemaker location. Therefore, careful considerations should also be given to the use of passive PBT for the treatment of patients with localized prostate cancer with pacemaker, as with the case in patients with thoracic and abdominal cancer.
